# Is resistant hypertension an independent predictor of all-cause mortality in individuals with type 2 diabetes? A prospective cohort study

**DOI:** 10.1186/s12916-019-1313-x

**Published:** 2019-04-25

**Authors:** Anna Solini, Giuseppe Penno, Emanuela Orsi, Enzo Bonora, Cecilia Fondelli, Roberto Trevisan, Monica Vedovato, Franco Cavalot, Olga Lamacchia, Marco G. Baroni, Antonio Nicolucci, Giuseppe Pugliese, for the Renal Insufficiency And Cardiovascular Events (RIACE) Study Group, Antonio Nicolucci, Giuseppe Pugliese, Lucilla Bollanti, Elena Alessi, Martina Vitale, Tiziana Cirrito, Paolo Cavallo-Perin, Gabriella Gruden, Bartolomeo Lorenzati, Franco Cavalot, Mariella Trovati, Leonardo Di Martino, Fabio Mazzaglia, Giampaolo Zerbini, Valentina Martina, Silvia Maestroni, Valentina Capuano, Emanuela Orsi, Eva Palmieri, Elena Lunati, Valeria Grancini, Veronica Resi, Antonio Pontiroli, Annamaria Veronelli, Barbara Zecchini, Maura Arosio, Laura Montefusco, Antonio Rossi, Guido Adda, Roberto Trevisan, Anna Corsi, Mascia Albizzi, Enzo Bonora, Giacomo Zoppini, Angelo Avogaro, Monica Vedovato, Giuseppe Penno, Laura Pucci, Daniela Lucchesi, Eleonora Russo, Monia Garofolo, Anna Solini, Francesco Dotta, Cecilia Fondelli, Laura Nigi, Susanna Morano, Tiziana Filardi, Irene Turinese, Marco Rossetti, Raffaella Buzzetti, Chiara Foffi, Mauro Cignarelli, Olga Lamacchia, Sabina Pinnelli, Lucia Monaco, Francesco Giorgino, Luigi Laviola, Annalisa Natalicchio, Giorgio Sesti, Francesco Andreozzi, Marco Giorgio Baroni, Giuseppina Frau, Alessandra Boi

**Affiliations:** 1 Department of Surgical, Medical, Molecular and Critical Area Pathology, University of Pisa, Pisa, Italy; 2 Department of Clinical and Experimental Medicine, University of Pisa, Pisa, Italy; 3 Diabetes Unit, IRCCS “Cà Granda - Ospedale Maggiore Policlinico” Foundation, Milan, Italy; 4 Division of Endocrinology, Diabetes and Metabolism, University and Hospital Trust of Verona, Verona, Italy; 5 Diabetes Unit, University of Siena, Siena, Italy; 6 Endocrinology and Diabetes Unit, Azienda Ospedaliera Papa Giovanni XXIII, Bergamo, Italy; 7 Department of Clinical and Experimental Medicine, University of Padua, Padua, Italy; 8 Department of Clinical and Biological Sciences, University of Turin, Orbassano, Italy; 9 Department of Medical Sciences, University of Foggia, Foggia, Italy; 10 Unit of Endocrinology and Diabetes, Department of Medical Sciences, University of Cagliari, Cagliari, Italy; 11 Centre for Outcomes Research and Clinical Epidemiology (CORESEARCH), Pescara, Italy; 12 Department of Clinical and Molecular Medicine, “La Sapienza” University, Via di Grottarossa, 1035-1039, 00189 Rome, Italy; 13 Present Address: Department of Experimental Medicine, “La Sapienza” University, Rome, Italy

**Keywords:** Resistant hypertension, Type 2 diabetes, All-cause mortality, Cardiovascular disease, Chronic kidney disease

## Abstract

**Background:**

Resistant hypertension is independently associated with an increased risk of death in the general hypertensive population. We assessed whether resistant hypertension is an independent predictor of all-cause mortality in individuals with type 2 diabetes from the Renal Insufficiency And Cardiovascular Events (RIACE) Italian Multicentre Study.

**Methods:**

On 31 October 2015, vital status information was retrieved for 15,656 of the 15,773 participants enrolled in 2006–2008. Based on baseline blood pressure (BP) values and treatment, participants were categorized as normotensive, untreated hypertensive, controlled hypertensive (i.e., on-target with < 3 drugs), uncontrolled hypertensive (i.e., not on-target with 1–2 drugs), or resistant hypertensive (i.e., uncontrolled with > 3 drugs or controlled with > 4 drugs). Kaplan–Meier and Cox proportional hazards regression analyses were used to assess the association with all-cause mortality.

**Results:**

Using the 130/80 mmHg targets for categorization, crude mortality rates and Kaplan–Meier estimates were highest among resistant hypertension participants, especially those with controlled resistant hypertension. As compared with resistant hypertension, risk for all-cause mortality was significantly lower for all the other groups, including individuals with controlled hypertension (hazard ratio 0.81 [95% confidence interval 0.74–0.89], *P* < 0.0001), but became progressively similar between resistant and controlled hypertension after adjustment for cardiovascular risk factors and complications/comorbidities. Also when compared with controlled resistant hypertension, mortality risk was significantly lower for all the other groups, including controlled hypertension, even after adjusting for cardiovascular risk factors (0.77 [0.63–0.95], *P* = 0.012), but not for complications/comorbidities (0.88 [0.72–1.08], *P* = 0.216). BP was well below target in the controlled hypertensive groups (resistant and non-resistant) and values < 120/70 mmHg were associated with an increased mortality risk. Results changed only partly when using the 140/90 mmHg targets for categorization.

**Conclusions:**

In the RIACE cohort, at variance with the general hypertensive population, resistant hypertension did not predict death beyond target organ damage. Our findings may be explained by the high mortality risk conferred by type 2 diabetes and the low BP values observed in controlled hypertensive patients, which may mask risk associated with resistant hypertension. Less stringent BP goals may be preferable in high-risk patients with type 2 diabetes.

**Trial registration:**

ClinicalTrials.gov, NCT00715481, retrospectively registered 15 July, 2008.

**Electronic supplementary material:**

The online version of this article (10.1186/s12916-019-1313-x) contains supplementary material, which is available to authorized users.

## Background

Type 2 diabetes is associated with excess mortality mainly, though not exclusively attributable to cardiovascular disease (CVD) [1]. The increased risk for CVD morbidity and mortality associated with type 2 diabetes requires a prompt recognition and management of the other comorbidities clustering with hyperglycemia and contributing to this high-risk profile, as demonstrated by the efficacy of multifactorial intervention in the Steno-2 study [2, 3]. In particular, control of hypertension represents a major issue, even though blood pressure (BP) targets are still a matter of debate, with recommended values ranging from < 130 to < 140 mmHg for systolic BP and from < 80 to < 90 mmHg for diastolic BP [4]. Unfortunately, though awareness and control of hypertension have improved in the last decades, a high percentage of diabetic hypertensive patients does not reach target BP levels [5, 6].

According to the 2008 Scientific Statement from the American Heart Association (AHA) [7], resistant hypertension is defined as uncontrolled BP despite the use of ≥ 3 anti-hypertensive medication classes or controlled BP while treated with ≥ 4 anti-hypertensive medication classes, with all agents prescribed at optimal dose amounts; ideally, one of these classes should be a diuretic. A recent revision of the AHA Scientific Statement has established that, in addition to a diuretic, the anti-hypertensive regimen should include also a long-acting calcium channel blocker (CCB) and a blocker of the renin-angiotensin system (RAS) [8]. Pooled data from North America and Europe indicated that 14.8% of treated hypertensive patients and 12.5% of all hypertensive individuals have resistant hypertension [9]. However, these prevalence estimates refer to “apparent resistant hypertension,” as population-based studies are unable to distinguish cases of “true resistant hypertension” from those of “pseudo-resistant hypertension,” i.e., individuals with “white coat” hypertension, non-adherence to medications, inappropriately prescribed anti-hypertensive regimen, and incorrect BP measurement due to cuff-related artifacts [10]. It has been estimated that individuals with pseudo-resistant hypertension are as many as those with true resistant hypertension [10], who however should include also patients with BP uncontrolled with < 2 drugs who would fail to achieve BP goal if treated with three drugs [11]. Subjects with resistant hypertension are usually older, more frequently obese and diabetic, and those with a higher prevalence of target organ damage, including CVD and chronic kidney disease (CKD), whereas data on gender and ethnicity are contrasting [7, 10–23].

A few longitudinal studies demonstrated that resistant hypertension is an independent predictor of all-cause and CVD mortality, CVD morbidity, and end-stage renal disease in the general hypertensive population, though different definitions of resistant and non-resistant hypertension were used [13–15, 18]. The increased risk remained after adjustment for several confounders, including CVD risk factors and target organ damage [13–15, 18]. In addition, some of these studies reported a worse prognosis in uncontrolled resistant hypertension (i.e., BP not on-target with ≥ 3 drugs) than in controlled resistant hypertension (i.e., BP on-target with ≥ 4 drugs) [13, 14, 18]. An independent association between resistant hypertension and adverse outcomes was also reported in hypertensive individuals with CVD [19–21] or CKD [22, 23].

Although the presence of diabetes has been invariably reported among predictors of adverse outcomes in hypertensive individuals [12–15, 18–22], only one study has reported a subgroup analysis in diabetic patients [18] and, so far, no study has evaluated the risk of death associated with resistant hypertension in a type 2 diabetes population. We have previously reported that prevalence of resistant hypertension was 17.4% among hypertensive individuals and 21.2% among treated hypertensive patients with type 2 diabetes from the Renal Insufficiency And Cardiovascular Events (RIACE) Italian Multicentre Study [24].

The present analysis aimed at assessing whether resistant hypertension at baseline is an independent predictor of subsequent death from any cause in individuals with type 2 diabetes from the RIACE cohort. To this end, individuals without hypertension or with non-resistant hypertension were compared with patients with resistant hypertension as reference group.

## Methods

### Design

The RIACE Italian Multicentre Study is an observational, prospective, cohort study on the impact of estimated glomerular filtration rate (eGFR) on morbidity and mortality in individuals with type 2 diabetes [25].

### Study population

The study population included 15,773 Caucasian patients (after excluding 160 individuals with missing or implausible values), consecutively attending 19 hospital-based, tertiary referral Diabetes Clinics of the National Health Service throughout Italy in the years 2006–2008. Exclusion criteria were dialysis or renal transplantation.

The vital status of the participants on 31 October 2015 was verified by interrogating the Italian Health Card database (http://sistemats1.sanita.finanze.it/wps/portal/), which provides updated and reliable information on all current Italian residents.

### Measurements

At baseline, study participants underwent a structured interview in order to collect the following information: age, smoking status, known diabetes duration, comorbidities, and current treatments.

Body mass index (BMI) was computed from weight and height, whereas waist circumference was calculated from log-transformed BMI values using sex-specific linear regression equations derived from waist measurements obtained in 4618 individuals. BP was measured with a sphygmomanometer after a 5-min rest. Two consecutive readings were taken 10 min apart by a trained observer with the patients seated with the arm at the heart level and the cuff correctly placed on the arm circumference. Standard adult cuffs were used (9–13 in.), except for severely obese patients, where large cuffs (13–17 in.) were employed. The second readings were used for the analysis [24]. Pulse pressure, a surrogate measure of arterial stiffness, was then calculated from systolic and diastolic BP values.

Triglycerides and total and HDL cholesterol were measured in fasting blood samples by colorimetric enzymatic method, and LDL cholesterol was calculated by the Friedewald formula. Hemoglobin (Hb) A_1c_ was measured by high-performance liquid chromatography using DCCT-aligned methods.

Diabetic kidney disease (DKD) was assessed based on albuminuria and eGFR. Albumin excretion rate (AER) was measured from 24-h urine collections or estimated from albumin-to-creatinine ratio in early-morning, first-voided urine samples, using a conversion formula developed in patients with type 1 diabetes [25, 26]. Albuminuria was measured in fresh urine samples by immunonephelometry or immunoturbidimetry. For each individual, one to three measurements were obtained; in cases of multiple measurements, the geometric mean was used for analysis. In subjects with multiple measurements (4062 with at least two and 2310 with three values), concordance rate between the first value and the geometric mean was > 90% for all albuminuria classes [26]. Serum (and urine) creatinine was measured by the modified Jaffe method, traceable to IDMS, and estimated eGFR was calculated by the CKD Epidemiology Collaboration equation [25]. Patients were then classified into the Kidney Disease: Improving Global Outcomes A1–A3 and G1–G5 categories and further stratified into the following DKD phenotypes, as previously reported [25]: no DKD (i.e., G1A1–G2A1), albuminuria alone (albuminuric DKD with preserved eGFR, i.e., G1A2–G2A2–G1A3–G2A3), reduced eGFR alone (nonalbuminuric DKD, i.e., G3A1–G4A1–G5A1), or albuminuria and reduced eGFR (albuminuric DKD with reduced eGFR, i.e., G3A2–G4A2–G5A2–G3A3–G4A3–G5A3).

In each center, presence of diabetic retinopathy (DR) was evaluated by an expert ophthalmologist by dilated fundoscopy. Based on the worst eye, individuals with mild or moderate non-proliferative DR were classified as having non-advanced DR, whereas those with severe non-proliferative DR, proliferative DR, or maculopathy were grouped into the advanced DR category, as previously reported [27].

Previous major acute CVD events (myocardial infarction, stroke, foot ulcer/gangrene/amputation, coronary, carotid, lower limb revascularization, and surgery for aortic aneurysm) were adjudicated based on hospital discharge records [28].

### Categorization of patients

Patients were stratified according to either the BP targets of < 130/80 mmHg, recommended for diabetic individuals at the time BP measures were obtained [29] and recently confirmed by the American College of Cardiology and AHA guidelines [30], or to the less stringent BP targets of < 140/90 mmHg, which are currently established by the American Diabetes Association, except for high-risk individuals [31]. The following groups were identified [24]: normotensive (NT); untreated hypertensive (UTHT); hypertensive on-target with 1, 2, or 3 drugs (controlled hypertension; CHT); hypertensive not on-target with 1 or 2 drugs (uncontrolled hypertension; UCHT); and hypertensive not on-target with > 3 drugs or on-target with > 4 drugs (resistant hypertension; RHT). The RHT group was further divided into two subgroups, based on whether patients were on-target with > 4 drugs (controlled resistant hypertension; CRHT) or were not on-target with > 3 drugs (uncontrolled resistant hypertension; UCRHT).

### Statistical analysis

Data are expressed as mean ± SD or median (interquartile range) for continuous variables, and number of cases and percentage for categorical variables. Comparisons among groups were performed by one-way ANOVA or Kruskal–Wallis test, according to the parametric or non-parametric distribution of continuous variables, followed by Bonferroni correction or Mann–Whitney test, respectively, for post hoc comparisons. The Pearson’s *χ*^2^ test was used for categorical variables.

Crude mortality rates were described as events per 1000 patient years, with 95% exact Poisson confidence intervals (CIs); death rates were also adjusted for age and gender by a Poisson regression model. Kaplan–Meier survival probabilities for all-cause mortality were estimated according to the above categorizations and differences were analyzed using the log-rank statistic. The hazard ratios (HRs) and their 95% CIs were estimated by Cox proportional hazards regression, unadjusted and adjusted for baseline age and gender (model 1); age, gender, and CVD risk factors, i.e., smoking status, diabetes duration, HbA_1c_, BMI, waist circumference, triglycerides, total and HDL cholesterol, and anti-hyperglycemic and lipid-lowering treatment (model 2); and age, gender, CVD risk factors, and complications/comorbidities, i.e., DKD phenotypes, DR grade, any CVD, and any cancer (model 3). In separate analyses, models were further adjusted for either BP or pulse pressure values at baseline to assess whether the excess risk associated with RHT was attributable to the higher levels of these parameters detected in RHT versus CHT participants. All the above analyses were repeated by including in the RHT group only patients on a diuretic or a CCB/RAS blocker/diuretic combination, according to the 2008 [7] and 2018 [8] definition of resistant hypertension, respectively. Finally, additional analyses, adjusted for age and gender, were performed to explore the relation between categories of on-treatment BP values and mortality. In all the above analyses, the RHT (or CRHT) group was used as reference to allow comparison with all other groups, i.e., NT and the various non-RHT groups (and UCRHT), thus distinguishing patients with CHT from those with UTHT or UCHT, who might include RHT individuals.

## Results

### Overall mortality in the study population

Valid information on vital status was retrieved for 15,656 participants (99.3% of the cohort). At the time of the census, 12,054 (77.0%) patients were alive, whereas 3602 (23.0%) individuals had died; death rate was 31.0 per 1000 person years (95% CI 30.0, 32.0) over a median follow-up of 8.0 years (interquartile range 7.5–8.5) [32, 33].

### Clinical features of the study population (based on the 130/80 mmHg BP targets)

The RIACE participants with RHT were 15.0% of the whole cohort (17.5% of all hypertensive individuals); of them, 13.5% were on-target with > 4 drugs (CRHT) and 86.5% were not on-target with > 3 drugs (UCRHT). As previously reported [24], RHT individuals were older, more often females and former smokers, and more frequently on insulin, lipid-lowering, anti-platelet, and anti-coagulant treatment, as compared to patients classified into the other groups. In addition, they had longer diabetes duration (except versus UCHT), lower eGFR, and higher BMI, waist circumference, triglycerides, albuminuria, and prevalence of DKD, advanced DR, and CVD (any and by vascular bed) (Table 1). Among RHT patients, CRHT participants had lower eGFR and HDL cholesterol and higher triglycerides and prevalence of CVD, driven by coronary events, and were more often on insulin and anti-coagulant therapy than UCRHT individuals, who were older and had higher total and LDL cholesterol (Additional file 1: Table S1).

**Table 1:** Baseline clinical features in the RIACE participants with valid information on vital status, stratified by BP status according to the 130/80 mmHg BP targets

Variable	NT	UTHT	CHT	UCHT	RHT	*P*
*n* (%)	2206 (14.09)	2378 (15.19)	3707 (23.68)	5014 (32.03)	2351 (15.02)	
Age, years	61.5 ± 11.7	64.3 ± 10.7	67.3 ± 10.0	68.0 ± 9.5	69.6 ± 8.7	< 0.0001
Gender, *n* (%)						< 0.0001
Females	833 (37.76)	948 (39.87)	1592 (42.95)	2270 (45.27)	1111 (47.26)	
Males	1373 (62.24)	1430 (60.13)	2115 (57.05)	2744 (54.73)	1240 (52.74)	
Smoking status, *n* (%)						< 0.0001
Never	1220 (55.30)	1352 (56.85)	2059 (55.54)	2890 (57.64)	1328 (56.49)	
Former	535 (24.25)	603 (25.36)	1084 (29.24)	1439 (28.70)	746 (31.73)	
Current	451 (20.44)	423 (17.79)	564 (15.21)	685 (13.66)	277 (11.78)	
Diabetes duration, years	10.7 ± 9.4	11.9 ± 9.7	13.3 ± 10.2	14.2 ± 10.4	14.4 ± 10.2	< 0.0001
HbA_1c_, mmol/mol	58.7 ± 17.5	58.9 ± 16.3	58.6 ± 16.8	59.1 ± 15.8	59.6 ± 16.3	< 0.0001
Anti-hyperglycemic treatment, *n* (%)						< 0.0001
Lifestyle	393 (17.82)	424 (17.83)	481 (12.98)	587 (11.71)	228 (9.70)	
Non-insulin	1310 (59.38)	1470 (61.82)	2244 (60.53)	3225 (64.32)	1370 (58.27)	
Insulin	503 (22.80)	484 (20.35)	982 (26.49)	1202 (23.97)	753 (32.03)	
BMI, kg/m^2^	27.29 ± 4.80	28.08 ± 4.72	29.08 ± 5.11	29.27 ± 5.09	30.61 ± 5.43	< 0.0001
Waist circumference, cm	99.1 ± 9.8	100.8 ± 9.6	102.7 ± 10.3	103.1 ± 10.3	105.8 ± 10.9	< 0.0001
Triglycerides, mmol/l	1.21 (0.88, 1.73)	1.28 (0.93, 1.83)	1.36 (0.98, 1.92)	1.36 (0.99, 1.90)	1.46 (1.07, 2.00)	< 0.0001
Total cholesterol, mmol/l	4.85 ± 0.96	4.94 ± 1.0	4.64 ± 1.0	4.80 ± 0.98	4.67 ± 0.96	< 0.0001
HDL cholesterol, mmol/l	1.31 ± 0.36	1.34 ± 0.37	1.25 ± 0.35	1.29 ± 0.35	1.26 ± 0.34	< 0.0001
LDL cholesterol, mmol/l	2.89 ± 0.82	2.93 ± 0.85	2.67 ± 0.84	2.80 ± 0.84	2.66 ± 0.81	< 0.0001
Lipid-lowering therapy, *n* (%)	671 (30.42)	779 (32.76)	1945 (52.47)	2468 (49.22)	1375 (58.49)	< 0.0001
Statins, *n* (%)	601 (27.24)	698 (29.35)	1804 (48.66)	2280 (45.47)	1271 (54.06)	< 0.0001
Anti-platelet therapy, *n* (%)	402 (18.22)	454 (19.09)	1876 (50.61)	2167 (43.22)	1349 (57.38)	< 0.0001
Anti-coagulant therapy, *n* (%)	25 (1.13)	31 (1.30)	226 (6.10)	180 (3.59)	207 (8.80)	< 0.0001
Albuminuria, mg/24 h	10.3 (5.5, 18.9)	11.1 (5,8, 21.4)	13.8 (6.6, 37.2)	14.4 (7.0, 36.5)	19.8 (9.1, 73.4)	< 0.0001
eGFR, ml·min^−1^·1.73 m^−2^	89.8 ± 18.4	86.9 ± 17.0	78.3 ± 21.3	78.6 ± 20.1	71.1 ± 22.6	< 0.0001
DKD phenotypes, *n* (%)						< 0.0001
Alb^−^/eGFR^−^	1776 (80.51)	1861 (78.26)	2213 (59.70)	3068 (61.19)	1066 (45.34)	
Alb^+^/eGFR^−^	275 (12.47)	352 (14.80)	737 (19.88)	1046 (20.86)	556 (23.65)	
Alb^−^/eGFR^+^	100 (4.53)	106 (4.46)	420 (11.33)	498 (9.93)	352 (14.97)	
Alb^+^/eGFR^+^	55 (2.49)	59 (2.48)	337 (9.09)	402 (8.02)	377 (16.04)	
DR, *n* (%)						< 0.0001
No	1883 (85.36)	1957 (82.30)	2862 (77.21)	3799 (75.77)	1688 (71.80)	
Non-advanced	193 (8.75)	245 (10.30)	496 (13.38)	683 (13.62)	327 (13.91)	
Advanced	127 (5.76)	176 (7.40)	349 (9.41)	532 (10.61)	336 (14.29)	
CVD, *n* (%)						
Any	214 (9.70)	253 (10.64)	1127 (30.40)	1196 (23.85)	830 (35.30)	< 0.0001
Acute myocardial infarction	67 (3.04)	68 (2.86)	627 (16.91)	525 (10.47)	455 (19.35)	< 0.0001
Coronary revascularization	63 (2.86)	69 (2.90)	588 (15.86)	468 (9.33)	391 (16.63)	< 0.0001
Any coronary event	107 (4.85)	113 (4.75)	830 (22.39)	756 (15.08)	590 (25.10)	< 0.0001
Stroke	28 (1.27)	38 (1.60)	147 (3.97)	173 (3.45)	127 (5.40)	< 0.0001
Carotid revascularization	54 (2.45)	72 (3.03)	227 (6.12)	313 (6.24)	190 (8.08)	< 0.0001
Any cerebrovascular event	79 (3.58)	109 (4.58)	346 (9.33)	458 (9.13)	300 (12.76)	< 0.0001
Ulcer/gangrene/amputation	50 (2.67)	54 (2.27)	154 (4.15)	175 (3.49)	123 (5.23)	< 0.0001
Lower limb revascularization	23 (1.04)	26 (1.09)	145 (3.91)	155 (3.09)	101 (4.30)	< 0.0001
Any peripheral event	67 (3.04)	75 (3.15)	258 (6.96)	288 (5.74)	195 (8.29)	< 0.0001
Aortic aneurysm	6 (0.27)	5 (0.21)	15 (0.40)	16 (0.32)	16 (0.68)	0.065
Cancer, *n* (%)	118 (5.35)	127 (5.34)	277 (7.47)	326 (6.50)	183 (7.78)	< 0.0001

Values are mean ± SD or median (interquartile range) for continuous variables, and number of cases (percentage) for categorical variables. *RIACE* Renal Insufficiency And Cardiovascular Events, *BP* blood pressure, *NT* normotension, *UTHT* untreated hypertension, *CHT* controlled hypertension (on-target with 1, 2, or 3 drugs), *UCHT* uncontrolled hypertension (not on-target with 1 or 2 drugs), *RHT* resistant hypertension (on-target with > 4 drugs or not on-target with > 3 drugs), *HbA*_*1c*_ hemoglobin A_1c_, *BMI* body mass index, *eGFR* estimated glomerular filtration rate, *DKD* diabetic kidney disease, *Alb*^*−*^*/eGFR*^*−*^ no DKD, *Alb*^*+*^*/eGFR*^*−*^ albuminuric DKD with preserved eGFR, *Alb*^*−*^*/eGFR*^*+*^ nonalbuminuric DKD, *Alb*^*+*^*/eGFR*^*+*^ albuminuric DKD with reduced eGFR, *DR* diabetic retinopathy, *CVD* cardiovascular disease

By definition, BP levels were higher in UTHT, UCHT, RHT, and UCRHT than in NT, CHT, and CRHT participants (Tables 2 and 3). Interestingly, values in CHT and CRHT individuals were well below 130/80 mmHg. Use of antihypertensive agents was significantly higher in RHT versus the other treated hypertensive groups for any class as well as in CRHT versus UCRHT individuals for RAS, α-, and β-blockers and diuretics (Henle’s loop and anti-aldosterone). Of note, pulse pressure was higher in UCHT, RHT, and, to a lesser extent, UTHT individuals versus the other groups and, within the RHT group, in UCRHT versus CRHT participants, as for BP values.

**Table 2:** BP values and anti-hypertensive treatment in the RIACE participants with valid information on vital status, stratified by BP status according to the 130/80 mmHg BP targets

Variable	NT	UTHT	CHT	UCHT	RHT	*P*
*n*, (%)	2206 (14.09)	2378 (15.19)	3707 (23.68)	5014 (32.03)	2351 (15.02)	
Systolic BP, mmHg	121.2 ± 8.4	145.6 ± 12.7	122.1 ± 8.4	149.4 ± 14.2	147.2 ± 17.5	< 0.0001
Diastolic BP, mmHg	73.7 ± 6.7	82.4 ± 8.4	73.1 ± 7.3	82.6 ± 9.2	80.5 ± 9.7	< 0.0001
Pulse pressure, mmHg	47.5 ± 8.5	63.2 ± 14.4	49.0 ± 8.8	66.8 ± 14.9	66.7 ± 16.7	< 0.0001
Number of anti-hypertensive agents	0 ± 0	0 ± 0	1.80 ± 0.77	1.48 ± 0.50	3.47 ± 0.65	< 0.0001
RAS blockers, *n* (%)	0 (0)	0 (0)	3064 (82.65)	3989 (79.56)	2287 (97.28)	< 0.0001
ACE-inhibitors, *n* (%)	0 (0)	0 (0)	2000 (53.95)	2651 (52.87)	1429 (60.78)	< 0.0001
ARBs, *n* (%)	0 (0)	0 (0)	1085 (29.27)	1346 (26.84)	1144 (48.66)	< 0.0001
Alpha-blockers, *n* (%)	0 (0)	0 (0)	200 (5.40)	240 (4.79)	507 (21.57)	< 0.0001
Beta-blockers, *n* (%)	0 (0)	0 (0)	871 (23.50)	749 (14.94)	1099 (46.75)	< 0.0001
Non-DHP CCBs, *n* (%)	0 (0)	0 (0)	247 (6.66)	285 (5.68)	257 (10.93)	< 0.0001
DHP CCBs, *n* (%)	0 (0)	0 (0)	707 (19.07)	870 (17.35)	1243 (52.87)	< 0.0001
Diuretics, *n* (%)	0 (0)	0 (0)	1410 (38.04)	1239 (23.71)	2013 (85.62)	< 0.0001
Thiazides, *n* (%)	0 (0)	0 (0)	826 (22.28)	795 (15.86)	1387 (59.00)	< 0.0001
Henle’s loop, *n* (%)	0 (0)	0 (0)	552 (14.89)	379 (7.56)	800 (34.03)	< 0.0001
Anti-aldosterone, *n* (%)	0 (0)	0 (0)	172 (4.64)	100 (1.99)	301 (12.8)	< 0.0001

Values are mean ± SD for continuous variables, unless otherwise specified. *RIACE* Renal Insufficiency And Cardiovascular Events, *BP* blood pressure, *NT* normotension, *UTHT* untreated hypertension, *CHT* controlled hypertension (on-target with 1, 2, or 3 drugs), *UCHT* uncontrolled hypertension (not on-target with 1 or 2 drugs), *RHT* resistant hypertension (on-target with > 4 drugs or not on-target with > 3 drugs), *RAS* renin-angiotensin system, *ACE* angiotensin-converting enzyme, *ARBs* angiotensin receptor blockers, *DHP* dihydropyridine, *CCBs* calcium channel blockers

**Table 3:** Baseline clinical features in the RIACE participants with valid information on vital status and resistant hypertension on-target with > 4 drugs or not on-target with > 3 drugs according to the 130/80 mmHg BP targets

Variables	CRHT	UCRHT	*P*
*n* (%)	305 (12.97)	2046 (87.03)	
Age, years	68.0 ± 8.8	69.8 ± 8.6	0.001
Gender, *n* (%)			0.136
Females	132 (43.28)	979 (47.85)	
Males	173 (56.72)	1067 (52.15)	
Smoking status, *n* (%)			0.226
Never	159 (52.13)	1169 (57.14)	
Former	104 (34.10)	642 (31.38)	
Current	42 (13.77)	235 (11.49)	
Diabetes duration, years	13.7 ± 9.8	14.5 ± 10.3	0.203
HbA_1c_, mmol/mol	60.7 ± 17.4	59.3 ± 16.1	0.208
Anti-hyperglycemic treatment, *n* (%)			0.008
Lifestyle	34 (11.15)	194 (9.48)	
Non-insulin	153 (50.16)	1217 (59.48)	
Insulin	118 (38.69)	635 (31.04)	
BMI, kg/m^2^	30.5 ± 5.6	30.6 ± 5.4	0.702
Waist circumference, cm	105.6 ± 11.1	105.8 ± 10.9	0.804
Triglycerides, mmol/l	1.54 (1.08, 2.17)	1.44 (1.07, 1.97)	0.041
Total cholesterol, mmol/l	4.50 ± 0.94	4.71 ± 0.97	< 0.0001
HDL cholesterol, mmol/l	1.15 ± 0.30	1.28 ± 0.35	< 0.0001
LDL cholesterol, mmol/l	2.54 ± 0.77	2.69 ± 0.83	0.003
Lipid-lowering therapy, *n* (%)	188 (61.64)	1187 (58.02)	0.231
Statins, *n* (%)	175 (57.38)	1096 (53.57)	0.213
Anti-platelet therapy, *n* (%)	189 (61.97)	1160 (56.70)	0.082
Anti-coagulant therapy, *n* (%)	47 (15.41)	160 (7.82)	< 0.0001
Albuminuria, mg/24 h	18.7 (9.2, 74.3)	20.0 (9.1, 72.9)	0.627
eGFR, ml·min^−1^·1.73 m^−2^	68.5 ± 24.1	71.5 ± 22.3	0.032
DKD phenotypes, *n* (%)			0.056
Alb^−^/eGFR^−^	127 (41.64)	939 (45.89)	
Alb^+^/eGFR^−^	63 (20.66)	493 (24.10)	
Alb^−^/eGFR^+^	57 (18.69)	295 (14.42)	
Alb^+^/eGFR^+^	58 (19.02)	319 (15.59)	
DR, *n* (%)			0.935
No	219 (71.80)	1469 (71.80)	
Non-advanced	44 (14.43)	283 (13.83)	
Advanced	42 (13.77)	294 (14.37)	
CVD, *n* (%)			
Any	136 (44.59)	694 (33.92)	< 0.0001
Acute myocardial infarction	90 (29.51)	365 (17.84)	< 0.0001
Coronary revascularization	78 (25.57)	313 (15.30)	< 0.0001
Any coronary event	115 (37.70)	475 (23.22)	< 0.0001
Stroke	10 (3.28)	117 (5.72)	0.079
Carotid revascularization	26 (8.52)	164 (8.02)	0.761
Any cerebrovascular event	35 (11.48)	265 (12.95)	0.471
Ulcer/gangrene/amputation	18 (5.90)	105 (5.13)	0.573
Lower limb revascularization	20 (6.56)	81 (3.96)	0.037
Any peripheral event	30 (9.84)	165 (8.06)	0.295
Aortic aneurysm	3 (0.98)	13 (0.64)	0.490
Cancer, *n* (%)	30 (9.84)	153 (7.48)	0.152
Systolic BP, mmHg	121.3 ± 8.8	151.0 ± 15.0	< 0.0001
Diastolic BP, mmHg	72.4 ± 7.1	81.7 ± 9.5	< 0.0001
Pulse pressure, mmHg	48.8 ± 9.0	69.3 ± 16.0	< 0.0001
Number of anti-hypertensive agents	4.21 ± 0.45	3.36 ± 0.60	< 0.0001
RAS blockers, *n* (%)	304 (99.67)	1983 (96.92)	0.006
ACE-inhibitors, *n* (%)	195 (63.93)	1234 (60.31)	0.227
ARBs, *n* (%)	151 (49.51)	993 (48.53)	0.751
Alpha-blockers, *n* (%)	91 (29.84)	416 (20.33)	< 0.0001
Beta-blockers, *n* (%)	223 (73.11)	876 (42.82)	< 0.0001
Non-DHP CCBs, *n* (%)	31 (10.16)	226 (11.05)	0.645
DHP CCBs, *n* (%)	169 (55.41)	1074 (52.49)	0.341
Diuretics, *n* (%)	287 (94.10)	1726 (84.36)	< 0.0001
Thiazides, *n* (%)	179 (58.69)	1208 (59.04)	0.907
Henle’s loop, *n* (%)	158 (51.80)	642 (31.38)	< 0.0001
Anti-aldosterone, *n* (%)	87 (28.52)	2046 (10.46)	< 0.0001

Values are mean ± SD or median (interquartile range) for continuous variables, and number of cases (percentage) for categorical variables. *RIACE* Renal Insufficiency And Cardiovascular Events, *CRHT* controlled resistant hypertension (on-target with > 4 drugs), *UCRHT* uncontrolled resistant hypertension (not on-target with > 3 drugs), *HbA*_*1c*_ hemoglobin A_1c_, *BMI* body mass index, *eGFR* estimated glomerular filtration rate, *DKD* diabetic kidney disease, *Alb*^*−*^*/eGFR*^*−*^ no DKD, *Alb*^*+*^*/eGFR*^*−*^ albuminuric DKD with preserved eGFR, *Alb*^*−*^*/eGFR*^*+*^ nonalbuminuric DKD, *Alb*^*+*^*/eGFR*^*+*^ albuminuric DKD with reduced eGFR, *DR* diabetic retinopathy, *CVD* cardiovascular disease, *BP* blood pressure, *RAS* renin-angiotensin system, *ACE* angiotensin-converting enzyme, *ARBs* angiotensin receptor blockers, *DHP* dihydropyridine, *CCBs* calcium channel blockers

### Association between resistant hypertension and mortality (based on the 130/80 mmHg BP targets)

**Fig. 1:**
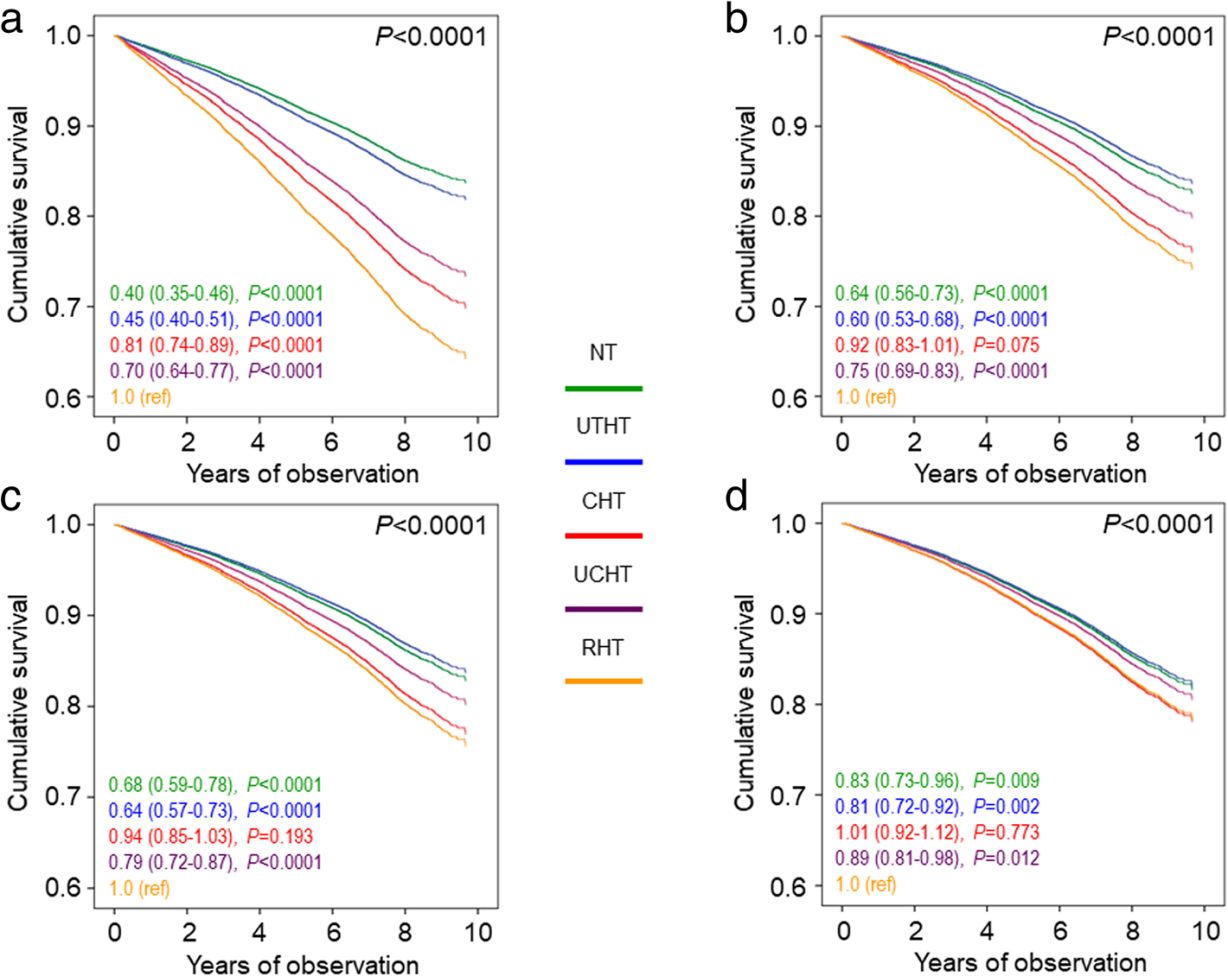
Cox proportional hazards regression, unadjusted (**a**) and adjusted for age and gender (**b**) plus CVD risk factors (**c**) plus complications/comorbidities (**d**), according to BP status (based on the 130/80 mmHg BP targets). HRs (95% CI) for mortality are shown for each group. BP = blood pressure; HR = hazard ratio; CI = confidence interval; NT = normotension (green); UTHT = untreated hypertension (blue); CHT = controlled hypertension (on-target with 1, 2, or 3 drugs, red); UCHT = uncontrolled hypertension (not on-target with 1 or 2 drugs, purple); RHT = resistant hypertension (on-target with > 4 drugs or not on-target with > 3 drugs, orange, reference)

**Fig. 2:**
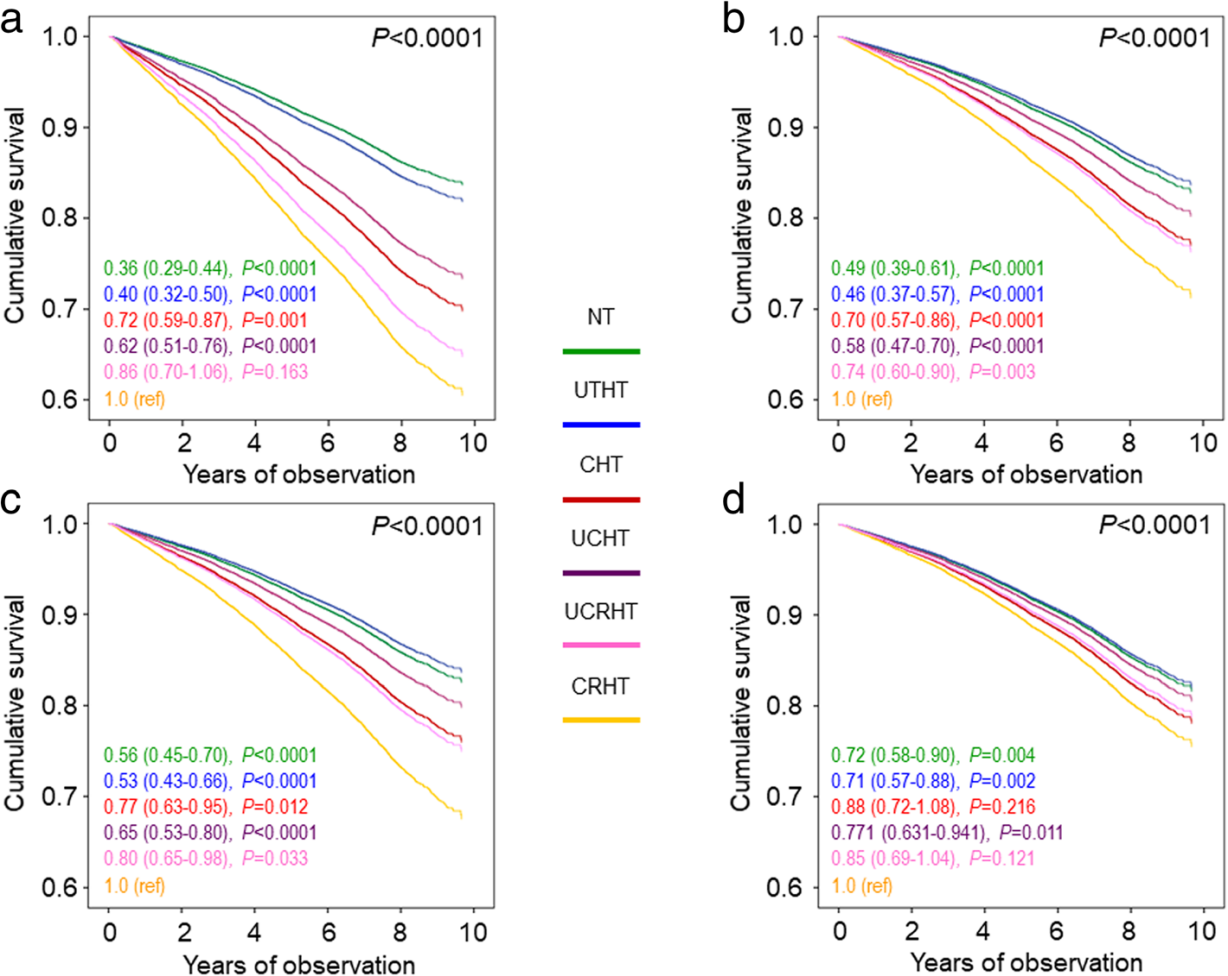
Cox proportional hazards regression, unadjusted (**a**) and adjusted for age and gender (**b**) plus CVD risk factors (**c**) plus complications/comorbidities (**d**), according to BP status (based on the 130/80 mmHg BP targets). HRs (95% CI) for mortality are shown for each group. BP = blood pressure; HR = hazard ratio; CI = confidence interval; NT = normotension (green); UTHT = untreated hypertension (blue); CHT = controlled hypertension (on-target with 1, 2, or 3 drugs, red); UCHT = uncontrolled hypertension (not on-target with 1 or 2 drugs, purple); UCRHT = uncontrolled resistant hypertension (not on-target with > 3 drugs, pink); CRHT = controlled resistant hypertension (on-target with > 4 drugs, orange, reference)

Crude mortality rates and Kaplan–Meier estimates were highest for RHT, intermediate for CHT and UCHT, and lowest for NT and UTHT participants (Table 4 and Additional file 2: Figure S1A). Differences in mortality rates (Table 4) were reduced after adjustment for age and gender. When compared to RHT, CHT was associated with a significantly lower risk of death only in the unadjusted analysis (HR 0.81 [95% CI 0.81–0.89], *P* < 0.0001) (Fig. 1a), whereas no difference was observed after adjustment for age and gender (model 1; Fig. 1b) and further adjustment for CVD risk factors (model 2; Fig. 1c) and complications/comorbidities (model 3; Fig. 1d). Interestingly, crude mortality rates (Table 4) and Kaplan–Meier estimates (not shown) were highest for CRHT. Differences in mortality rates were attenuated after adjustment for age and gender (Table 4). As compared with CRHT individuals, unadjusted HRs (Fig. 2a) were significantly lower in all other groups (except UCRHT), including CHT participants (0.72 [0.59–0.87], *P* = 0.001). Differences between RHT and CHT were maintained after adjustment for age and gender (0.70 [0.57–0.86], *P* < 0.0001) (Fig. 2b), were attenuated when adjusting also for CVD risk factors (0.77 [0.63–0.95], *P* = 0.012) (Fig. 2c), and disappeared when accounting for complications/comorbidities (0.88 [0.72–1.08], *P* = 0.216) (Fig. 2d). No change was observed when further adjusting for BP or pulse pressure values or when only RHT individuals on a diuretic or a CCB/RAS blocker/diuretic combination were included in the analysis (not shown).

**Table 4:** Mortality rates in the RIACE participants with valid information on vital status, stratified by BP status according to the 130/80 mmHg or 140/90 mmHg BP targets

	*N*	Events	Percent events	Events per 1000 patient-years (95% CI) unadjusted	*P*	Events per 1000 patient-years (95% CI) age- and gender-adjusted	*P*
Study groups							
*130/80 mmHg BP targets*					< 0.0001		< 0.0001
NT	2206	316	14.32	18.36 (16.33–20.38)		10.15 (8.64–11.92)	
UTHT	2378	380	15.98	20.65 (18.57–22.73)		9.52 (8.17–11.10)	
CHT	3707	980	26.44	36.67 (34.37–38.96)		14.39 (12.61–16.41)	
UCHT	5014	1180	23.53	31.76 (29.94–33.57)		11.93 (10.50–13.55)	
RHT	2351	746	31.73	44.94 (41.72–48.17)		15.68 (13.70–17.94)	
CRHT	305	107	35.08	50.93 (41.28–60.58)		20.36 (16.34–25.38)	
UCRHT	2046	639	31.23	44.08 (40.66–47.49)		15.07 (13.13–17.30)	
*140/90 mmHg BP targets*					< 0.0001		< 0.0001
NT	3445	492	14.28	18.31 (16.69–19.93)		9.72 (8.39–11.26)	
UTHT	1139	204	17.91	23.31 (20.11–26.51)		9.86 (8.24–11.80)	
CHT	6298	1619	25.71	35.44 (33.72–37.17)		13.61 (12.01–15.43)	
UCHT	2952	707	23.95	32.26 (29.88–34.64)		11.82 (10.32–13.53)	
RHT	1882	580	31.83	43.56 (40.01–47.10)		15.82 (13.76–18.18)	
CRHT	559	173	30.95	43.93 (37.38–50.47)		17.15 (14.23–20.67)	
UCRHT	1263	407	32.22	45.46 (41.04–49.88)		15.31 (13.18–17.77)	

*RIACE* Renal Insufficiency And Cardiovascular Events, *BP* blood pressure, *CI* confidence interval; *NT* normotension, *UTHT* untreated hypertension, *CHT* controlled hypertension (on-target with 1, 2, or 3 drugs), *UCHT* uncontrolled hypertension (not on-target with 1 or 2 drugs), *RHT* resistant hypertension (on-target with > 4 drugs or not on-target with > 3 drugs), *CRHT* controlled resistant hypertension (on-target with > 4 drugs), *UCRHT* uncontrolled resistant hypertension (not on-target with > 3 drugs)

### Clinical features of the study population (based on the 140/90 mmHg BP targets)

When the cohort was stratified according to the 140/90 mmHg BP targets, the percentage of individuals with RHT decreased (11.6% of the whole cohort and 14.9% of all hypertensive individuals), with a higher proportion of CRHT (30.7%), but the distribution of clinical parameters among study groups and subgroups did not change appreciably (Additional file 1: Tables S1 and Additional file 3: Table S2). However, the average BP values of the controlled hypertensive groups (CHT and CRHT) became closer to 130/80 mmHg, as several individuals with values between 130 and 139 and/or 80–89 mmHg, formerly assigned to the UCHT and UCRHT categories, respectively, were included in these groups.

### Association between resistant hypertension and mortality (based on the 140/90 mmHg BP targets)

Crude and age- and gender-adjusted mortality rates, Kaplan–Meier estimates, and HRs were similar to those observed when participants were stratified according to the more stringent BP targets (Table 4, Additional file 2: Figure S1B and Additional file 4: Figure S2), except that (a) CHT was associated with a significantly lower risk of death than RHT both in the unadjusted (0.78 [0.71–0.86], *P* < 0.0001) and the adjusted (model 1: 0.86 [0.78–0.94], *P* = 0.001, and model 2: 0.90 [0.81–0.98], *P* = 0.022, but not model 3: 0.99 [0.90–1. 08], *P* = 0.755) analysis; and (b) the HRs for the CRHT and UCRHT subgroups did not diverge appreciably (Additional file 5: Figure S3).

### Association between on-treatment BP values and mortality

**Fig. 3:**
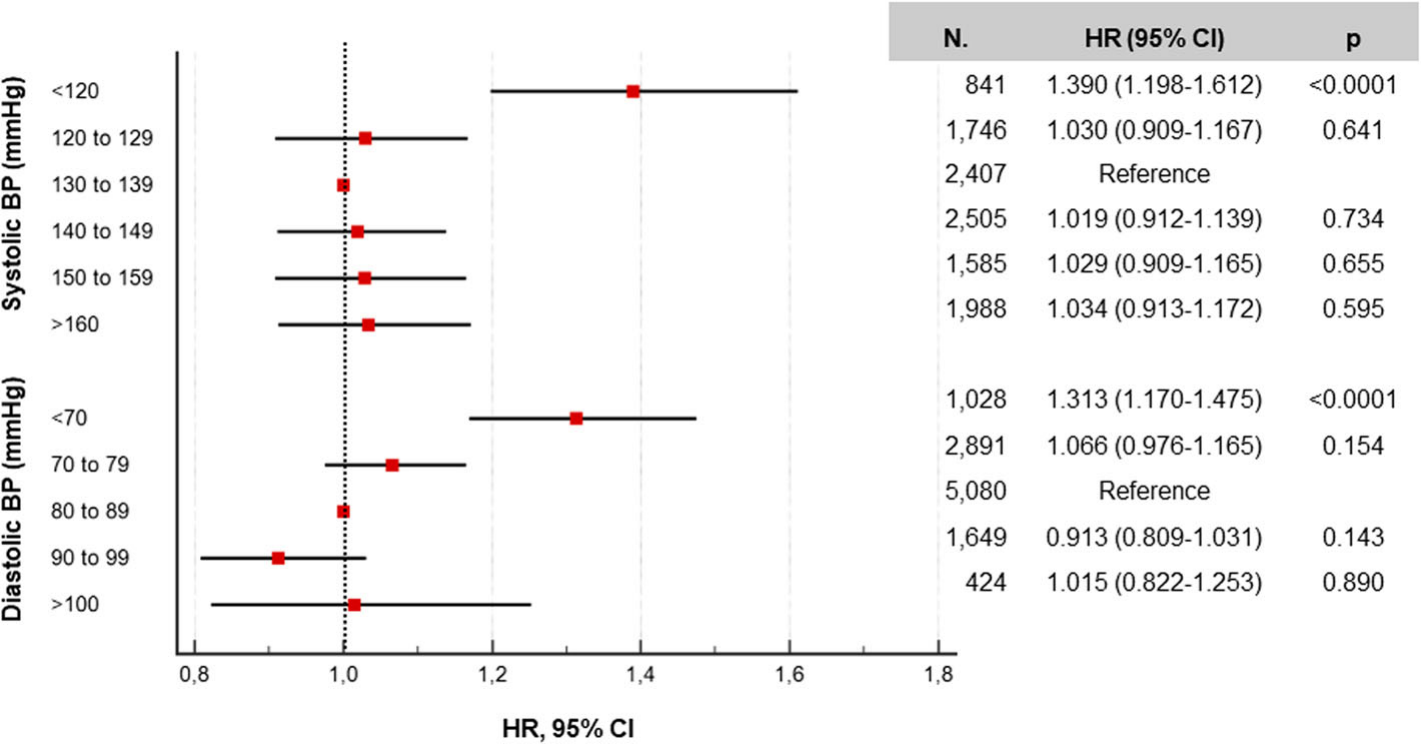
Age- and gender-adjusted HRs (95% CI) for mortality according to systolic (A) and diastolic (B) BP categories, regardless of group stratification

There was a U-shape association between on-treatment BP values and all-cause mortality. In particular, using the 130–139 mmHg category of systolic BP and the 80–89 mmHg category of diastolic BP as reference, risk of death increased for systolic BP < 120 mmHg and diastolic BP < 70 mmHg, but not for values above 140 and 90 mmHg, respectively, consistent with the previously reported inverse association of mortality with systolic and diastolic BP in this cohort (Fig. 3) [32].

## Discussion

This analysis of the RIACE cohort of patients with type 2 diabetes shows that resistant hypertension was associated with an increased risk of all-cause mortality, which however was progressively attenuated after adjustment for confounders. In particular, using the 130/80 mmHg targets, an increased risk of death in individuals with resistant versus controlled hypertension was observed only in the unadjusted analysis, whereas, using the 140/90 mmHg targets, it was maintained also in the adjusted analysis, except when accounting for complications/comorbidities. These observations indicate that CVD risk profile and particularly complications (indicating target organ damage) and comorbidities, which are significantly worse in individuals with resistant hypertension, drive the increased risk of death associated with this condition compared to controlled hypertension. However, our finding that, in type 2 diabetes, resistant hypertension does not predict death beyond target organ damage is at odds with data from the general hypertensive population [12–15, 18–21] and hypertensive individuals with CVD [19, 20] or CKD [22, 23]. Indeed, in these studies, diabetes was found to be an independent correlate of adverse outcomes, suggesting that it poses a significantly greater risk of death masking that associated with resistant hypertension. This interpretation is consistent with a subgroup analysis of the Antihypertensive and Lipid-Lowering Treatment to Prevent Heart Attack Trial (ALLHAT) cohort, showing a significant association of resistant hypertension with all-cause mortality in non-diabetic, but not in diabetic individuals [18].

Another intriguing observation coming from our data is that, among resistant hypertensive patients, those on-target with > 4 drugs showed a higher mortality risk than individuals not on-target with > 3 drugs. Again, this finding is at variance with data from the general hypertensive population [13, 14] and also with a retrospective analysis of a group of US Veterans with resistant hypertension, showing that controlling BP values resulted in lower mortality compared with individuals who remained uncontrolled over a 6-year follow-up [34]. However, in these studies, the BP values in participants with controlled resistant hypertension were higher than in our cohort, at least when the RIACE participants were categorized using the 130/80 mmHg targets, i.e., when differences in mortality between the two resistant hypertensive subgroups were actually observed. This suggests that our unexpected finding may be explained, at least partly, by the quite low BP levels observed in controlled resistant hypertensive individuals. Indeed, also patients with non-resistant controlled hypertension showed BP values well below target when using the 130/80 mmHg goals for categorization, thus suggesting that low BP levels might also contribute to explain the observation that risk of death in these individuals was not significantly lower than in patients with resistant hypertension in the adjusted analyses. This interpretation is supported by the higher mortality risk associated with lower BP values regardless of group assignment, which is consistent with the J-curve phenomenon occurring in high-risk patients, such as those with established CVD, CKD, and/or diabetes. In these individuals, an impaired blood flow auto-regulation would elevate the BP threshold below which organ perfusion is reduced [35]. This J-curve effect has been described in several post hoc analyses of intervention trials in which however reverse causality could not be excluded and was indeed suggested by the evidence of a similar phenomenon in the placebo-treated groups that calls into question its clinical relevance [35]. Data from the Taipei City Geriatric Health Examination Database [36], the CLARIFY registry [37], and a cohort of US Veterans [38] showed that low BP values are indeed associated with increased mortality in community-dwelling older adults and in individuals with CVD and CKD. A previous study on hypertensive individuals with manifest vascular disease reported a somewhat higher increase in all-cause and CVD mortality risk versus controlled hypertension in subjects with controlled resistant hypertension than in those with uncontrolled resistant hypertension [21] and another survey in hypertensive patients with atherothrombosis showed that those poorly controlled on ≥ 3 agents had an increased risk of stroke and congestive heart failure, whereas those on ≥ 4 anti-hypertensive agents (irrespective of BP control) had an increased risk of all adverse outcomes, including all-cause mortality, except stroke [39]. Though not originally designed to address this issue, our study provides further support to the existence of a clinically meaningful J-curve effect, which may have increased mortality risk among individuals with controlled hypertension, thus masking the excess risk associated with resistant hypertension.

A major strength of this study is that it is the first analyzing a type 2 diabetes population. Other strengths include the large sample size, the long-term follow-up, the low number of participants lost to follow-up, and the separation of individuals with untreated or uncontrolled hypertension from those with controlled hypertension among participants without resistant hypertension. In fact, patients with untreated or uncontrolled hypertension were likely those with a recent diagnosis of hypertension or not adequately treated, respectively. Presumably, they have subsequently received a treatment or a more aggressive one, thus experiencing a reduction of BP levels, though some of them may have fallen into the resistant hypertensive category. This would explain the relatively low mortality rate in the untreated and, to a lesser extent, uncontrolled hypertensive individuals, despite BP levels being similar to those of patients with resistant hypertension, and also the finding that mortality risk did not increase significantly with higher BP levels.

Among the limitations, lack of availability of multiple BP measurements over time may have resulted in a misclassification bias, as also some of the normotensive, controlled hypertensive, and resistant hypertensive participants may have switched to another BP status category during the follow-up. Moreover, we acknowledge that true treatment-resistant hypertension may have been misclassified with pseudo-resistance in a number of cases, as we could not assess adherence and appropriate prescription of anti-hypertensive therapy and to perform ambulatory BP monitoring, the gold standard method for excluding white coat hypertension. Indeed, ambulatory, but not office BP was shown to be associated with CVD morbidity and mortality in subjects with resistant hypertension, thus highlighting the confounding role of pseudo-resistant hypertension [40]. Another limitation is that not all the patients classified as resistant hypertensives were on a diuretic or a diuretic/CCB/RAS blocker combination, though results did not change when only individuals meeting these criteria were considered. This finding is consistent with the results of the ALLHAT, in which prognoses in patients with resistant hypertension were similar across treatment groups, though participants assigned to chlorthalidone were less likely to develop this condition [41]. Moreover, though use of anti-aldosterone therapy is not an absolute criterion, prevalence of resistant hypertension in our cohort might have been overestimated also because of the low proportion of resistant hypertensive patients receiving this treatment (12.8 and 13.9%, according to the 130/80 and 140/90 mmHg BP targets, respectively), despite evidence that spironolactone is effective in reducing BP in resistant hypertensive individuals [42], including those with diabetes [43]. Our finding that use of anti-aldosterone agents was significantly more frequent in controlled than uncontrolled individuals with resistant hypertension militates in favor of this interpretation. A further limitation is that our main finding that resistant hypertension is not an independent predictor of death beyond target organ damage cannot be generalized until validated in at least one independent type 2 diabetes population. Finally, the observational design of the study makes causal interpretation impossible*.*

## Conclusions

In individuals with type 2 diabetes from the RIACE cohort, resistant hypertension did not predict death beyond the increased burden of target organ damage characterizing this condition. In addition, risk of death was higher in individuals with controlled resistant hypertension than in those with uncontrolled resistant hypertension.

Both these findings are at variance with data from the general hypertensive population and require confirmation in other cohorts of patients with type 2 diabetes. They may be related to the high mortality risk conferred by type 2 diabetes as well as to the detrimental effect of the low BP values detected in individuals with controlled hypertension (resistant and non-resistant), which may have masked the increased risk associated with resistant hypertension per se. The demonstration of a J-curve phenomenon in our cohort further supports the concept that less stringent BP goals may be preferable in individuals with type 2 diabetes, especially in those at high CVD and renal risk, though this issue is still a matter of debate.

## Additional files

Additional file 1: **Table S1.** Baseline clinical features in the RIACE participants with valid information on vital status, stratified by BP status according to the 140/90 mmHg BP targets. (DOCX 17 kb)

Additional file 2: **Figure S1.** Cumulative survival by Kaplan Meier analysis according to BP status, based on the 130/80 mmHg (A) and 140/90 mmHg (B) BP targets. Numbers (percentages) of death are shown for each group. NT = normotension; UTHT = untreated hypertension; CHT = controlled hypertension; UCHT = uncontrolled hypertension; RHT = resistant hypertension. (DOC 824 kb)

Additional file 3: **Table S2.** Baseline clinical features in the RIACE participants with valid information on vital status and resistant hypertension on-target with > 4 drugs or not on-target with > 3 drugs according to the 140/90 mmHg BP targets. (DOCX 16 kb)

Additional file 4: **Figure S2.** Cox proportional hazards regression, unadjusted (A) and adjusted for age and gender (B) plus CVD risk factors (C) plus complications/comorbidities (D), according to BP status (based on the 140/90 mmHg BP targets). HRs (95% CI) for mortality are shown for each group. NT = normotension; UTHT = untreated hypertension; CHT = controlled hypertension; UCHT = uncontrolled hypertension; RHT = resistant hypertension. (DOC 809 kb)

Additional file 5: **Figure S3.** Cox proportional hazards regression, unadjusted (A) and adjusted for age and gender (B) plus CVD risk factors (C) plus complications/comorbidities (D), according to BP status (based on the 140/90 mmHg BP targets). HRs (95% CI) for mortality are shown for each group. NT = normotension; UTHT = untreated hypertension; CHT = controlled hypertension; UCHT = uncontrolled hypertension; CRHT = controlled resistant hypertension; UCRHT = uncontrolled resistant hypertension. (DOC 1012 kb)

## Abbreviations

AER: Albumin excretion rate
AHA: American Heart Association
ALLHAT: Antihypertensive and Lipid-Lowering Treatment to Prevent Heart Attack Trial
BMI: Body mass index
BP: Blood pressure
CCB: Calcium channel blocker
CHT: Controlled hypertension
CIs: Confidence intervals
CKD: Chronic kidney disease
CRHT: Controlled resistant hypertension
CVD: Cardiovascular disease
DKD: Diabetic kidney disease
DR: Diabetic retinopathy
eGFR: Estimated glomerular filtration rate
HbA_1c_: Hemoglobin A_1c_
HRs: Hazard ratios
NT: Normotensive
RAS: Renin-angiotensin system
RHT: Resistant hypertension
RIACE: Renal Insufficiency And Cardiovascular Events
UCHT: Uncontrolled hypertension
UCRHT: Uncontrolled resistant hypertension
UTHT: Untreated hypertensive
