# Supplementary material for: Is resistant hypertension an independent predictor of all-cause mortality in individuals with type 2 diabetes? A prospective cohort study
Source: BMC Med. 2019 Apr 25;17:83. doi: 10.1186/s12916-019-1313-x (PMC6482506; doi:10.1186/s12916-019-1313-x)
Supplement: Supplementary file 1 — Table S1. Baseline clinical features in the RIACE participants with valid information on vital status, stratified by BP status according to the 140/90 mmHg BP targets. (DOCX 17 kb) [file 12916_2019_1313_MOESM1_ESM.docx]

**Additional file 1: Table S1.** Baseline clinical features in the RIACE participants with valid information on vital status, stratified by BP status according to the 140/90 mmHg BP targets.

| Variable | NT | UTHT | CHT | UCHT | RHT | *P* |
| --- | --- | --- | --- | --- | --- | --- |
| n (%) | 3,445 (22.00) | 1,139 (7.28) | 6,298 (40.23) | 2,952 (18.86) | 1,822 (11.64) |  |
| Age, years | 62.0±11.5 | 65.8±10.0 | 67.5±9.8 | 68.4±9.3 | 69.5±8.6 | <0.0001 |
| Gender, n (%) |  |  |  |  |  | <0.0001 |
| Females | 1,302 (37.79) | 479 (42.05) | 2,749 (43.65) | 1,359 (46.04) | 865 (47.48) |  |
| Males | 2,143 ((62.21)) | 660 (57.95) | 3,549 (56.35) | 1,593 (53.96) | 957 (52.52) |  |
| Smoking status, n (%) |  |  |  |  |  | <0.0001 |
| Never | 1,940 (56.31) | 632 (55.49) | 3,524 (55.95) | 1,720 (58.27) | 1,033 (56.70) |  |
| Former | 826 (23.98) | 312 (27.39) | 1,848 (29.34) | 855 (28.96) | 566 (31.06) |  |
| Current | 679 (19.71) | 195 (17.12) | 926 (14.70) | 377 (12.77) | 223 (12.24) |  |
| Diabetes duration, years | 10.9±9.3 | 12.8±10.2 | 13.3±10.1 | 14.8±10.6 | 14.7±10.3 | <0.0001 |
| HbA_1c_, mmol/mol | 58.3±16.8 | 60.4±16.8 | 58.6±16.4 | 59.6±15.8 | 59.9±16.1 | <0.0001 |
| Anti-hyperglycaemic treatment, n (%) |  |  |  |  |  | <0.0001 |
| Lifestyle | 615 (17.85) | 202 (17.73) | 796 (12.64) | 333 (11.28) | 167 (9.17) |  |
| Non-insulin | 2,093 (60.75) | 687 (60.32) | 3,889 (61.75) | 1,897 (64.26) | 1,053 (57.79) |  |
| Insulin | 737 (21.39) | 250 (21.95) | 1,613 (25.61) | 722 (24.46) | 602 (33.04) |  |
| BMI, kg/m^2^ | 27.5±4.7 | 28.3±4.8 | 29.2±5.1 | 29.3±5.2 | 30.7±5.5 | <0.0001 |
| Waist circumference, cm | 99.6±9.7 | 101.2±9.8 | 103.0±10.2 | 103.2±10.5 | 105.9±11.1 | <0.0001 |
| Triglycerides, mmol/l | 1.21 (0.88, 1.74) | 1.33 (0.95, 1.90) | 1.37 (0.99, 1.92) | 1.36 (0.98, 1.90) | 1.46 (1.08, 2.02) | <0.0001 |
| Total cholesterol, mmol/l | 4.87±0.97 | 5.03±1.01 | 4.70±0.99 | 4.84±1.00 | 4.67±0.95 | <0.0001 |
| HDL cholesterol, mmol/l | 1.32±0.36 | 1.35±0.37 | 1.27±0.35 | 1.30±0.35 | 1.26±0.35 | <0.0001 |
| LDL cholesterol, mmol/l | 2.90±0.83 | 2.97±0.87 | 2.72±0.84 | 2.82±0.85 | 2.65±0.80 | <0.0001 |
| Lipid-lowering therapy, n (%) | 1,066 (30.94) | 384 (33.71) | 3,268 (51.89) | 1,451 (49.15) | 1,069 (58.67) | <0.0001 |
| Statins, n (%) | 953 (27.7) | 346 (30.4) | 3,016 (47.9) | 1,343 (45.5) | 996 (54,7) | <0.0001 |
| Anti-platelet therapy, n (%) | 637 (18.49) | 219 (19.23) | 3,113 (49.43) | 1,228 (41.60) | 1,051 (57.68) | <0.0001 |
| Anti-coagulant therapy, n (%) | 43 (1.25) | 13 (1.14) | 347 (5.51) | 101 (3.42) | 165 (9.06) | <0.0001 |
| Albuminuria, mg/24 hours | 10.4 (5.5, 19.0) | 11.8 (6.0, 24.3) | 14.2 (6.9, 37.0) | 14.4 (6.9, 38.4) | 20.2 (9.3, 83.1) | <0.0001 |
| eGFR, ml·min^-1^·1.73m^-2^ | 89.4±17.9 | 85.1±16.9 | 78.0±21.1 | 78.5±19.8 | 70.5±22.7 | <0.0001 |
| DKD phenotypes, n (%) |  |  |  |  |  | <0.0001 |
| Alb^-^/eGFR^-^ | 2,789 (80.96) | 848 (74.45) | 3,755 (59.62) | 1,796 (60.84) | 796 (43.69) |  |
| Alb^+^/eGFR^-^ | 431 (12.51) | 196 (17.21) | 1,271 (20.18) | 627 (21.24) | 441 (24.20) |  |
| Alb^-^/eGFR^+^ | 147 (4.27) | 59 (5.18) | 703 (11.16) | 295 (9.99) | 272 (14.93) |  |
| Alb^+^/eGFR^+^ | 78 (2.26) | 36 (3.16) | 569 (9.03) | 234 (7.93) | 313 (17.18) |  |
| DR, n (%) |  |  |  |  |  |  |
| No | 2,944 (85.46) | 896 (78.67) | 4,878 (77.45) | 2,183 (73.95) | 1,288 (70.69) |  |
| Non-advanced | 316 (9.17) | 125 (10.97) | 814 (12.92) | 424 (14.36) | 268 (14.71) |  |
| Advanced | 185 (5.37) | 118 (10.36) | 606 (9.62) | 345 (11.69) | 266 (14.60) |  |
| CVD, n (%) |  |  |  |  |  |  |
| Any | 333 (9.67) | 134 (11.76) | 1,811 (28.76) | 694 (23.51) | 648 (35.57) | <0.0001 |
| Acute myocardial infarction | 103 (2.99) | 32 (2.81) | 982 (15.59) | 264 (8.94) | 361 (19.81) | <0.0001 |
| Coronary revascularization | 97 (2.82) | 35 (3.07) | 892 (14.16) | 247 (8.37) | 308 (16.90) | <0.0001 |
| Any coronary event | 163 (4.73) | 57 (5.00) | 1,309 (20.78) | 401 (13.58) | 466 (25.58) | <0.0001 |
| Stroke | 50 (1.45) | 16 (1.40) | 257 (4.08) | 93 (3.15) | 97 (5.32) | <0.0001 |
| Carotid revascularization | 87 (2.53) | 39 (3.42) | 378 (6.00) | 206 (6.98) | 146 (8.01) | <0.0001 |
| Any cerebro-vascular event | 134 (3.89) | 54 (4.74) | 595 (9.45) | 280 (9.49) | 229 (12.57) | <0.0001 |
| Ulcer/gangrene/amputation | 71 (2.06) | 33 (2.90) | 233 (3.70) | 118 (4.00) | 101 (5.54) | <0.0001 |
| Lower limb revascularization | 38 (1.10) | 11 (0.97) | 225 (3.57) | 96 (3.25) | 80 (4.39) | <0.0001 |
| Any peripheral event | 100 (2.90) | 42 (3.69) | 400 (6.35) | 185 (6.27) | 156 (8.56) | <0.0001 |
| Aortic aneurysm | 8 (0.23) | 3 (0.26) | 26 (0.41) | 9 (0.30) | 12 (0.66) | <0.0001 |
| Cancer, n (%) | 174 (5.05) | 71 (6.23) | 477 (7.57) | 178 (6.03) | 131 (7.19) | <0.0001 |
| Systolic BP, mmHg | 126.7±10.5 | 155.4±10.9 | 128.4±10.3 | 157.9±12.4 | 149.7±19.1 | <0.0001 |
| Diastolic BP, mmHg | 76.5±7.8 | 83.6±9.4 | 76.0±8.1 | 84.3±9.9 | 80.7±10.2 | <0.0001 |
| Pulse pressure, mmHg | 50.2±9.9 | 71.9±13.3 | 52.5±10.0 | 73.6±14.0 | 69.0±17.7 | <0.0001 |
| Number of anti-hypertensive agents | 0±0 | 0±0 | 1.79±0.77 | 1.49±0.50 | 3.61±0.68 | <0.0001 |
| RAS blockers, n (%) | 0 (0) | 0 (0) | 5,191 (82.42) | 2,371 (80.32) | 1,778 (97.59) | <0.0001 |
| ACE-inhibitors, n (%) | 0 (0) | 0 (0) | 3,430 (54.46) | 1,534 (51.96) | 1,116 (61.25) | <0.0001 |
| ARBs, n (%) | 0 (0) | 0 (0) | 1,812 (28.77) | 847 (28.69) | 916 (50.27) | <0.0001 |
| Alpha-blockers, n (%) | 0 (0) | 0 (0) | 367 (5.83) | 149 (5.05) | 431 (23.66) | <0.0001 |
| Beta-blockers, n (%) | 0 (0) | 0 (0) | 1,379 (21.90) | 429 (14.53) | 911 (50.00) | <0.0001 |
| Non-DHP CCBs, n (%) | 0 (0) | 0 (0) | 434 (6.89) | 149 (5.05) | 206 (11.31) | <0.0001 |
| DHP CCBs, n (%) | 0 (0) | 0 (0) | 1,276 (20.26) | 548 (18.56) | 996 (54.67) | <0.0001 |
| Diuretics, n (%) | 0 (0) | 0 (0) | 2,358 (37.44) | 722 (24.46) | 1,582 (86.83) | <0.0001 |
| Thiazides, n (%) | 0 (0) | 0 (0) | 1,438 (22.83) | 472 (15.99) | 1,098 (60.26) | <0.0001 |
| Henle loop, n (%) | 0 (0) | 0 (0) | 870 (13.81) | 208 (7.05) | 653 (35.84) | <0.0001 |
| Anti-aldosterone, n (%) | 0 (0) | 0 (0) | 259 (4.11) | 61 (2.07) | 253 (13.89) | <0.0001 |

Values are mean±SD or median (interquartile range) for continuous variables, and number of cases (percentage) for categorical variables. RIACE = Renal Insufficiency And Cardiovascular Events; BP = blood pressure; NT = normotension; UTHT = untreated hypertension; CHT = controlled hypertension (on target with 1,2 or 3 drugs); UCHT = uncontrolled hypertension (not on-target with 1 or 2 drugs); RHT = resistant hypertension (on-target with >4 drugs or not on-target with >3 drugs); HbA_1c_ = haemoglobin A_1c_; BMI = body mass index; eGFR = estimated glomerular filtration rate; DKD = diabetic kidney disease; Alb^-^/eGFR^-^ = no DKD; Alb^+^/eGFR^-^ = albuminuric DKD with preserved eGFR; Alb^-^/eGFR^+^ = nonalbuminuric DKD; Alb^+^/eGFR^+^ = albuminuric DKD with reduced eGFR; DR = diabetic retinopathy; CVD = cardiovascular disease; BP = blood pressure; RAS = renin-angiotensin system; ACE = angiotensin-converting enzyme; ARBs = angiotensin receptor blockers; DHP = dihydropyridine; CCBs = calcium channel blockers.
