# Supplementary material for: Is resistant hypertension an independent predictor of all-cause mortality in individuals with type 2 diabetes? A prospective cohort study
Source: BMC Med. 2019 Apr 25;17:83. doi: 10.1186/s12916-019-1313-x (PMC6482506; doi:10.1186/s12916-019-1313-x)
Supplement: Supplementary file 2 — Figure S1. Cumulative survival by Kaplan Meier analysis according to BP status, based on the 130/80 mmHg (A) and 140/90 mmHg (B) BP targets. Numbers (percentages) of death are shown for each group. NT = normotension; UTHT = untreated hypertension; CHT = controlled hypertension; UCHT = uncontrolled hypertension; RHT = resistant hypertension. (DOC 824 kb) [file 12916_2019_1313_MOESM2_ESM.doc]

**
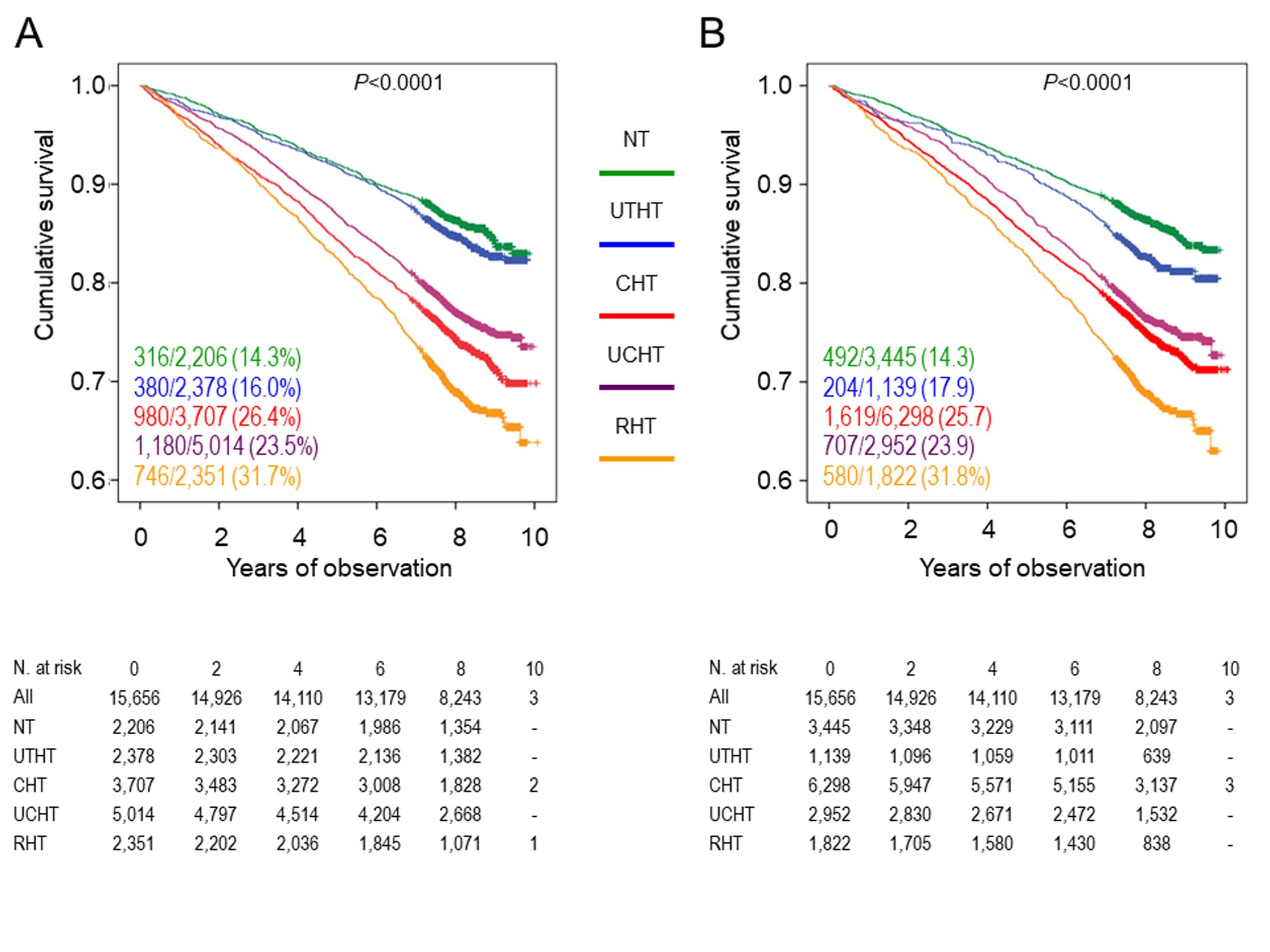
**

**Additional file 2: Fig. S1.** Cumulative survival by Kaplan Meier analysis according to BP status, based on the 130/80 mmHg (A) and 140/90 mmHg (B) BP targets. Numbers (percentages) of death are shown for each group. BP = blood pressure; NT = normotension (green); UTHT = untreated hypertension (blue); CHT = controlled hypertension (on target with 1,2 or 3 drugs, red); UCHT = uncontrolled hypertension (not on-target with 1 or 2 drugs, purple); RHT = resistant hypertension (on-target with >4 drugs or not on-target with >3 drugs, orange).
