# Supplementary material for: Is resistant hypertension an independent predictor of all-cause mortality in individuals with type 2 diabetes? A prospective cohort study
Source: BMC Med. 2019 Apr 25;17:83. doi: 10.1186/s12916-019-1313-x (PMC6482506; doi:10.1186/s12916-019-1313-x)
Supplement: Supplementary file 3 — Table S2. Baseline clinical features in the RIACE participants with valid information on vital status and resistant hypertension on-target with > 4 drugs or not on-target with > 3 drugs according to the 140/90 mmHg BP targets. (DOCX 16 kb) [file 12916_2019_1313_MOESM3_ESM.docx]

**Additional file 3: Table S2.** Baseline clinical features in the RIACE participants with valid information on vital status and resistant hypertension on-target with >4 drugs or not-on target with >3 drugs according to the 140/90 mmHg BP targets.

| Variables | CRHT | UCRHT | *P* |
| --- | --- | --- | --- |
| n (%) | 559 (30.7) | 1,263 (69.3) |  |
| Age, years | 68.1±8.9 | 70.1±8.4 | <0.0001 |
| Gender, n (%) |  |  | 0.143 |
| Females | 251 (44.9) | 614 (48.6) |  |
| Males | 308 (55.1) | 957 (51.4) |  |
| Smoking status, n (%) |  |  | 0.071 |
| Never | 298 (53.3) | 735 (58.2) |  |
| Former | 180 (32.2) | 386 (30.6) |  |
| Current | 81 (14.5) | 142 (11.2) |  |
| Diabetes duration, years | 13.9±9.8 | 15.0±10.5 | 0.034 |
| HbA_1c_, mmol/mol | 7.64±1.51 | 7.62±1.45 | 0.829 |
| Anti-hyperglycaemic treatment, n (%) |  |  | 0.211 |
| Lifestyle | 54 (9.7) | 113 |  |
| Non-insulin | 306 (54.7) | 747 (59.1) |  |
| Insulin | 199 (35.6) | 403 (31.9) |  |
| BMI, kg/m^2^ | 31.0±5.5 | 30.5±5.5 | 0.088 |
| Waist circumference, cm | 106.7±11.1 | 105.6±11.1 | 0.055 |
| Triglycerides, mmol/l | 1.47 (1.08-2.09) | 1.46 (1.08-1.99) | 0.245 |
| Total cholesterol, mmol/l | 4.55±0.92 | 4.73±0.95 | <0.0001 |
| HDL cholesterol, mmol/l | 1.19±0.32 | 1.29±0.35 | <0.0001 |
| LDL cholesterol, mmol/l | 2.59±0.77 | 2.68±0.80 | 0.026 |
| Lipid-lowering therapy, n (%) | 349 (62.4) | 720 (57.0) | 0.030 |
| Statins, n (%) | 329 (58.9) | 667 (52.8) | 0.017 |
| Anti-platelet therapy, n (%) | 351 (62.8) | 700 (55.4) | 0.003 |
| Anti-coagulant therapy, n (%) | 68 (12.2) | 97 (7.7) | 0.002 |
| Albuminuria, mg/24 hours | 18.5 (8.7-82.9) | 21.0 (9.5-84.2) | 0.857 |
| eGFR, ml·min^-1^·1.73m^-2^ | 70.5±24.0 | 70.5±22.1 | 0.956 |
| DKD phenotypes, n (%) |  |  | 0.318 |
| Alb^-^/eGFR^-^ | 250 (44.7) | 546 (43.2) |  |
| Alb^+^/eGFR^-^ | 123 (22.0) | 318 (25.2) |  |
| Alb^-^/eGFR^+^ | 93 (16.6) | 179 (14.2) |  |
| Alb^+^/eGFR^+^ | 93 (16.6) | 220 (17.4) |  |
| DR, n (%) |  |  | 0.401 |
| No | 406 (72.6) | 882 (69.8) |  |
| Non-advanced | 80 (14.3) | 188 (14.9) |  |
| Advanced | 73 (13.1) | 193 (15.3) |  |
| CVD, n (%) |  |  |  |
| Any | 236 (42.2) | 412 (32.6) | <0.0001 |
| Acute myocardial infarction | 153 (27.4) | 208 (16.5) | <0.0001 |
| Coronary revascularization | 130 (23.3) | 178 (14.1) | <0.0001 |
| Any coronary event | 195 (34.9) | 271 (21.5) | <0.0001 |
| Stroke | 23 (4.1) | 74 (5.9) | 0.126 |
| Carotid revascularization | 44 (7.9) | 102 (8.1) | 0.882 |
| Any cerebro-vascular event | 64 (11.4) | 165 (13.1) | 0.338 |
| Ulcer/gangrene/amputation | 30 (5.4) | 71 (5.6) | 0.826 |
| Lower limb revascularization | 31 (5.5) | 49 (3.9) | 0.109 |
| Any peripheral event | 49 (8.8) | 107 (8.5) | 0.836 |
| Aortic aneurysm | 13 (0.64) | 3 (0.98) | 0.490 |
| Cancer, n (%) | 43 (7.7) | 88 (7.0) | 0.581 |
| Systolic BP, mmHg | 128.2±10.8 | 159.3±13.2 | <0.0001 |
| Diastolic BP, mmHg | 75.6±8.3 | 83.0±10.2 | <0.0001 |
| Pulse pressure, mmHg | 52.6±11.0 | 76.3±15.0 | <0.0001 |
| Number of anti-hypertensive agents | 4.2±0.5 | 3.4±0.6 | <0.0001 |
| RAS blockers, n (%) | 555 (99.3) | 1,223 (96.8) | 0.002 |
| ACE-inhibitors, n (%) | 370 (66.2) | 746 (59.1) | 0.004 |
| ARBs, n (%) | 283 (50.6) | 633 (50.1) | 0.842 |
| Alpha-blockers, n (%) | 158 (28.3) | 273 (21.6) | 0.002 |
| Beta-blockers, n (%) | 382 (68.3) | 529 (41.9) | <0.0001 |
| Non-DHP CCBs, n (%) | 59 (10.6) | 147 (11.6) | 0.500 |
| DHP CCBs, n (%) | 316 (56.5) | 680 (53.8) | 0.288 |
| Diuretics, n (%) | 523 (93.6) | 1,059 (83.8) | <0.0001 |
| Thiazides, n (%) | 275 (49.2) | 378 (29.9) | <0.0001 |
| Henle’s loop, n (%) | 342 (61.2) | 756 (59.9) | 0.595 |
| Anti-aldosterone, n (%) | 137 (24.5) | 116 (9.2) | <0.0001 |

Values are mean±SD or median (interquartile range) for continuous variables, and number of cases (percentage) for categorical variables. RIACE = Renal Insufficiency And Cardiovascular Events; CRHT = controlled resistant hypertension (on-target with >4 drugs); UCRHT = uncontrolled resistant hypertension (not on-target with >3 drugs); HbA_1c_ = haemoglobin A_1c_; BMI = body mass index; eGFR = estimated glomerular filtration rate; DKD = diabetic kidney disease; Alb^-^/eGFR^-^ = no DKD; Alb^+^/eGFR^-^ = albuminuric DKD with preserved eGFR; Alb^-^/eGFR^+^ = nonalbuminuric DKD; Alb^+^/eGFR^+^ = albuminuric DKD with reduced eGFR; DR = diabetic retinopathy; CVD = cardiovascular disease; BP = blood pressure; RAS = renin-angiotensin system; ACE = angiotensin-converting enzyme; ARBs = angiotensin receptor blockers; DHP = dihydropyridine; CCBs = calcium channel blockers.
